# A genetic variant of CYP2R1 identified in a cat with type 1B vitamin D-dependent rickets: a case report

**DOI:** 10.1186/s12917-019-1784-1

**Published:** 2019-02-18

**Authors:** Takahiro Teshima, Sena Kurita, Takashi Sasaki, Hirotaka Matsumoto, Ayaka Niina, Daijiro Abe, Nobuo Kanno, Hidekazu Koyama

**Affiliations:** 1 Laboratory of Veterinary Internal Medicine, Department of Veterinary Clinical Medicine, School of Veterinary Medicine, Faculty of Veterinary Science, Nippon Veterinary and Life Science University, 1-7-1 Kyonan-cho, Musashino, Tokyo, 180-8602 Japan; 2 Laboratory of Veterinary Clinical Pathology, Department of Veterinary Clinical Medicine, School of Veterinary Medicine, Faculty of Veterinary Science, Nippon Veterinary and Life Science University, 1-7-1 Kyonan-cho, Musashino, Tokyo, 180-8602 Japan; 3 Animal Research Center, Sapporo Medical University School Medicine, S-1 W-17, Chuo-ku, Sapporo, Hokkaido 060-8556 Japan; 4 Veterinary Medical Teaching Hospital, Nippon Veterinary and Life Science University, 1-7-1 Kyonan-cho, Musashino, Tokyo, 180-8602 Japan; 5 Laboratory of Veterinary Surgery, Department of Veterinary Clinical Medicine, School of Veterinary Medicine, Faculty of Veterinary Science, Nippon Veterinary and Life Science University, 1-7-1 Kyonan-cho, Musashino, Tokyo, 180-8602 Japan

**Keywords:** Calcitriol, DNA sequence, Frameshift mutation, Hypocalcemia, Vitamin D deficiency, Vitamin D-dependent rickets

## Abstract

**Background:**

Vitamin D-dependent rickets is rare in animals and humans. Several types of this condition are associated with genetic variants related to vitamin D metabolism. This is the first report of type 1B vitamin D-dependent rickets in a cat.

**Case presentation:**

Here, we describe the case of a 3-month-old female domestic short-haired cat previously fed on commercial kitten food that presented at our clinic with seizures, lethargy, and generalized pain. Serum and ionized calcium concentrations and 1,25-dihydroxycholecalciferol in this cat were low, and radiographs showed skeletal demineralization and abnormally wide growth plates on the long bones. Initially, simple vitamin D deficiency was suspected; however, the cat’s profile, which included fed a well-balanced commercial diet, together with the findings of additional laboratory tests and the cat’s unresponsiveness to various treatments, raised the suspicion of vitamin D-dependent rickets. Examination of the DNA sequences of *CYP2R1* and *CYP27B1* genes, which are genes linked with vitamin D metabolism, showed a *CYP2R1* frameshift mutation in exon 5 (where T is deleted at position c.1386). This mutation alters the amino acid sequence from position 462, while the stop codon introduced at position 481 prematurely truncates the 501 amino acid full-length protein. With this knowledge, a new treatment regime based on a standard dose of calcitriol was started and this markedly improved the cat’s condition.

**Conclusions:**

To the best of our knowledge, the present case is the first description of type 1B vitamin D-dependent rickets linked with a genetic variant of *CYP2R1* in a cat.

**Electronic supplementary material:**

The online version of this article (10.1186/s12917-019-1784-1) contains supplementary material, which is available to authorized users.

## Background

**Fig. 1:**
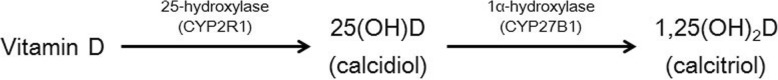
Vitamin D metabolism pathway in cats

Hypocalcemia and skeletal abnormalities can occur in cats as a result of primary hypoparathyroidism, nutritional secondary hyperparathyroidism, renal secondary hyperparathyroidism, intestinal malabsorption, or vitamin D-dependent rickets (VDDR), but the latter disease is rare in cats. In domestic cats, vitamin D3 is only acquired from dietary sources, and a two-step enzymatic pathway is required for its conversion to the active form by the same process that occurs with human vitamin D3 (calcitriol) (Fig. 1) [1]. In the liver, vitamin D 25-hydroxylase (CYP2R1) catalyzes the initial hydroxylation of vitamin D (cholecalciferol) to 25-hydroxycholecalciferol (calcidiol). In the kidneys, 1-alpha-hydroxylase (CYP27B1) then catalyzes the hydroxylation and metabolic activation of calcidiol to hormonally active 1,25-hydroxycholecalciferol (calcitriol). Calcitriol binds to and activates the nuclear vitamin D receptor (VDR), which regulates calcium homeostasis. Mutations in the *CYP27B1*, *CYP2R1*, and *VDR* genes result in types 1A, 1B, and 2 VDDR, respectively, in humans [2]. Feline cases of VDDR have been characterized, and the treatment strategies for it documented, but the causal mutations have rarely been determined [3–9]. In veterinary medicine, only two cats were previously identified with a genetic defect resulting in feline type 1A VDDR [4, 5], and type 1B VDDR has not been reported previously in a cat.

## Case presentation

A 3-month-old 1.1 kg female domestic short-haired cat presented with a 3-week history of seizure and generalized pain. The cat had been fed on a commercial kitten food. The cat was examined by the referring veterinarian because of its seizures and reluctance to move for 2 weeks before its initial presentation. The cat’s serum biochemistry profile revealed hypocalcemia (total calcium 1.27 mmol/L; reference range 1.97–2.82 mmol/L) and high alkaline phosphatase (835 U/L; reference range 14–192 U/l), aspartate transaminase (70 U/L; reference range 0–32 U/L), total bilirubin (3.4 mg/dL; reference range 0–0.9 mg/dL), and creatine kinase (3470 U/L; reference range 0–394 U/L). Urea and creatinine were within the reference range (Table 1). The complete blood count revealed no abnormalities, and the tests results for feline leukemia virus antigen and antibodies against feline immunodeficiency virus were negative. Based on these results, the referring veterinarian suspected that the cause of the seizures was related to hypocalcemia. Therefore, the cat was treated with levetiracetam (20 mg/kg, IV, BID) and calcium gluconate 8.5% (0.5 ml/kg, PO, BID) for 2 weeks, but its clinical condition did not improve.

**Table 1:** Serum biochemistry profile from the referring veterinarian

	value	reference range
Total protein (g/L)	67	52–82
Albumin (g/L)	29	22–39
Sodium (mmol/L)	158	150–165
Potassium (mmol/L)	4.9	3.7–4.9
Chloride (mmol/L)	118	115–126
Urea Nitrogen (mg/dL)	19	16–33
Creatinine (mg/dL)	53	53–141
Calcium (mmol/L)	1.27	1.97–2.82
Inorganic phosphorus (mmol/L)	2.55	1.45–2.65
Glucose (mmol/L)	6.44	4.31–8.57
Alanine transaminase (U/L)	80	12–115
Aspartate transaminase (U/L)	70	0–32
Alkaline phosphatase (U/L)	835	14–192
Total biliirubin (mg/dL)	3.4	0–0.9
Creatine kinase (U/L)	3470	0–394

**Fig. 2:**
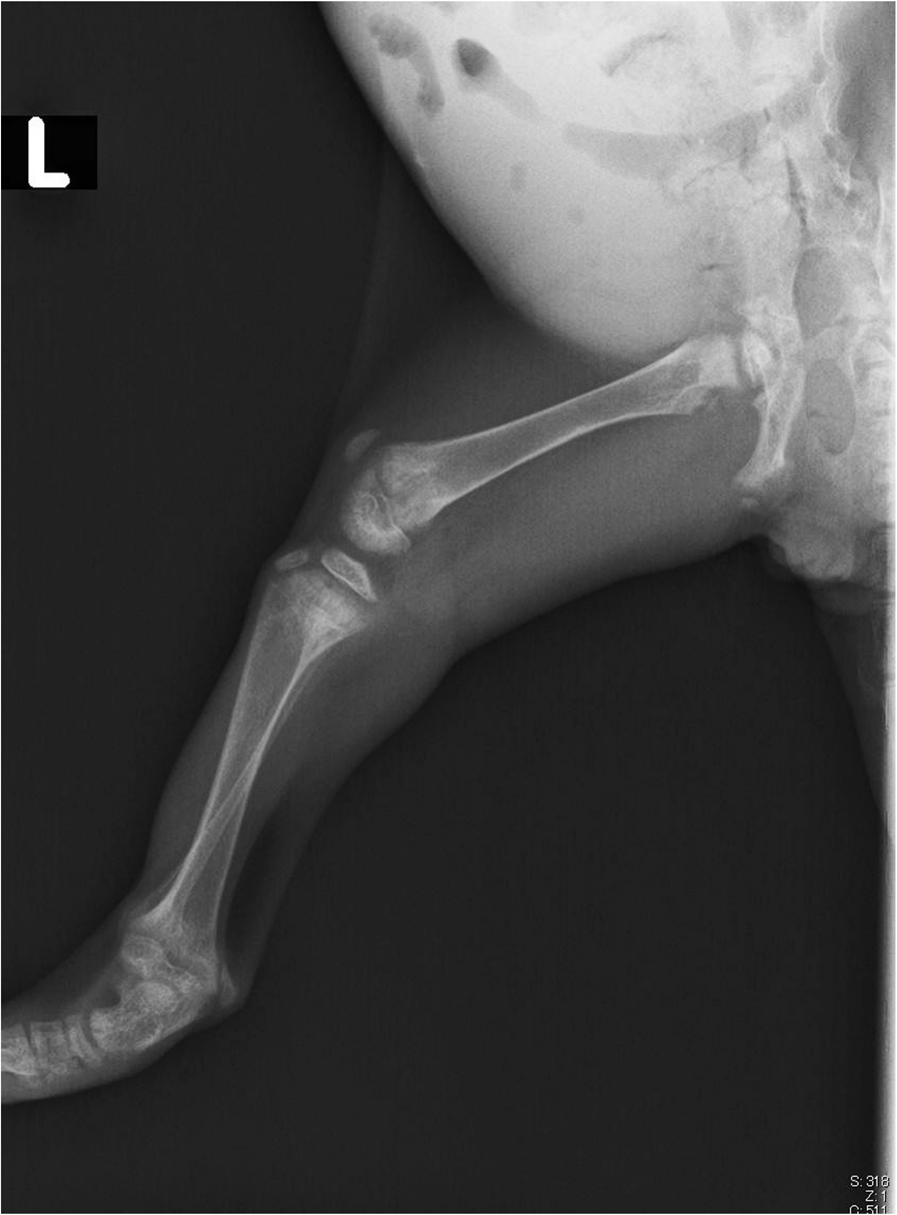
Skeletal demineralization and abnormally wide growth plates in the left hindlimb of the cat at 3 months of age

On initial examination, the cat was lethargic and reluctant to move. Her appetite was good, but defecation appeared to be painful. Her family history, including whether the parents and littermates were alive, was unknown. Serum biochemistry revealed that both the total and ionized calcium levels were low (total calcium 1.55 mmol/L; reference range 2.05–3.02 mmol/L, ionized calcium 0.74 mmol/L; reference range 1.20–1.35 mmol/L). Radiographs showed skeletal demineralization and the presence of abnormally wide growth plates on the long bones (Fig. 2). The differential diagnosis for hypocalcemia with skeletal abnormalities includes primary hypoparathyroidism, nutritional secondary hyperparathyroidism, renal secondary hyperparathyroidism, intestinal malabsorption, and VDDR. However, the cat had previously eaten a well-balanced commercial kitten food (Science Diet kitten, Hill’s Colgate Japan) before becoming obviously ill, so nutritional deficiency seemed highly unlikely. The levels of parathyroid hormone (Immulyze intact PTH III, Simens) and 1,25-dihydroxycholecalciferol (1,25(OH)2D RIA Kit FR, Immunodiagnostic Systems) were determined as elements of the differential diagnosis of hypocalcemia. The parathyroid hormone level was high (99.7 pg/ml; reference range 8.0–25.0 pg/ml), but 1,25-dihydroxycholecalciferol (23.1 pg/ml) was low compared with the levels of healthy cats (Table 2). The urinalysis revealed a specific gravity of 1.032 and traces of protein. No bacterial growth was detected in the urine culture. Consequently, VDDR was suspected to be the cause of hypocalcemia and skeletal abnormalities in this cat.

**Table 2:** Comparison of serum and ionized calcium, parathyroid hormone, and 1,25-dihydroxycholecalciferol levels

		Affected cat	Healthy cat		
	Reference range		6-month-old, intact female	6-month-old, intact male	9-month-old, spayed female
Serum calcium (mmol/L)	2.05–3.02	1.62	2.52	2.40	2.34
Ionized calcium (mmol/L)	1.20–1.35	0.74	1.31	1.26	1.29
Parathyroid hormone (pg/mL)	8.0–25.0	99.7	8.7	9.9	9.2
25-hydroxycholecalciferol (nmol/L)	―	12	133	148	126
1,25-dihydroxycholecalciferol (pg/mL)	―	23.1	232	251	227

On the day after the initial examination, the cat experienced seizures suspected of being hypocalcemic in origin. Slow administration of calcium gluconate 8.5% IV (1 ml/kg) was commenced, and the cat was continuously monitored by ECG, but no arrhythmia was detected. Several calcium gluconate 8.5% (1 ml/kg, IV) treatment stopped the seizures, but neither her total calcium nor her ionized calcium level increased sufficiently. Therefore, the cat was administered 8.5% calcium gluconate (starting dose, 1.0 ml/kg/h) in 0.9% saline solution by continuous rate infusion (CRI) (Table 3).

**Table 3:** Serum biochemistry and the doses of the vitamin D analogue and calcium gluconate administered during treatment

Day	Serum calcium (reference range: 2.05–3.02 mmol/L)	Ionized calcium (reference range: 1.20–1.35 mmol/L)	Serum inorganic phosphorus (reference range: 0.84–1.94 mmol/L)	Serum alkaline phosphatase (reference range: 38–165 U/L)	Serum creatine kinase (reference range: 89–312 U/L)	Calcium gluconate 8.5% treatment (ml/kg/h CRI)	Alphacarcidiol treatment (μg/kg PO)	Calcitriol treatment (ng/kg PO)
1	1.55	0.80	2.10	2262	2462	1.0 ml/kg IV several times		―
2	1.05	0.42	2.91	―	―	1.0	0.03 q8h	―
3	1.62	0.76	―	―	―	3.0	0.03 q8h	―
4	1.45	0.81	2.00	1386	1729	3.0	0.03 q8h	―
5	1.87	―	―	―	―	2.5	0.06 q8h	―
6	2.45	―	―	―	―	2.2	0.06 q8h	―
7	2.17	―	―	―	―	2.0	0.09 q8h	―
8	2.15	0.84	―	―	―	1.8	0.09 q8h	―
9	2.10	―	1.91	358	295	1.4	0.12 q8h	―
10	1.90	―	―	―	―	1.0	0.15 q8h	―
11	1.85	0.93	2.13	315	―	1.0	0.37 q8h	―
12	1.82	―	―	―	―	Discontinued	0.56 q8h	―
13	2.10	―	―	―	―	―	0.56 q8h	―
14	2.17	―	―	―	―	―	1.00 q8h	―
15	2.10	―	―	―	―	―	1.00 q8h	―
16	2.17	0.88	1.94	―	―	―	1.00 q8h	―
28	1.87	0.84	2.00	1615	907	―	1.00 q8h	―
47	2.05	―	2.07	1436	275	―	1.00 q12h	―
82	2.35	―	1.94	848	―	―	1.00 q12h	―
121	2.30	1.02	1.87	335	218	―	Discontinued	3.5 q24h
142	2.17	0.97	1.71	297	284	―	―	3.0 q24h
186	2.55	1.27	1.78	―	―	―	―	2.8 q24h
207	2.64	1.27	1.69	―	―	―	―	2.6 q24h

**Fig. 3:**
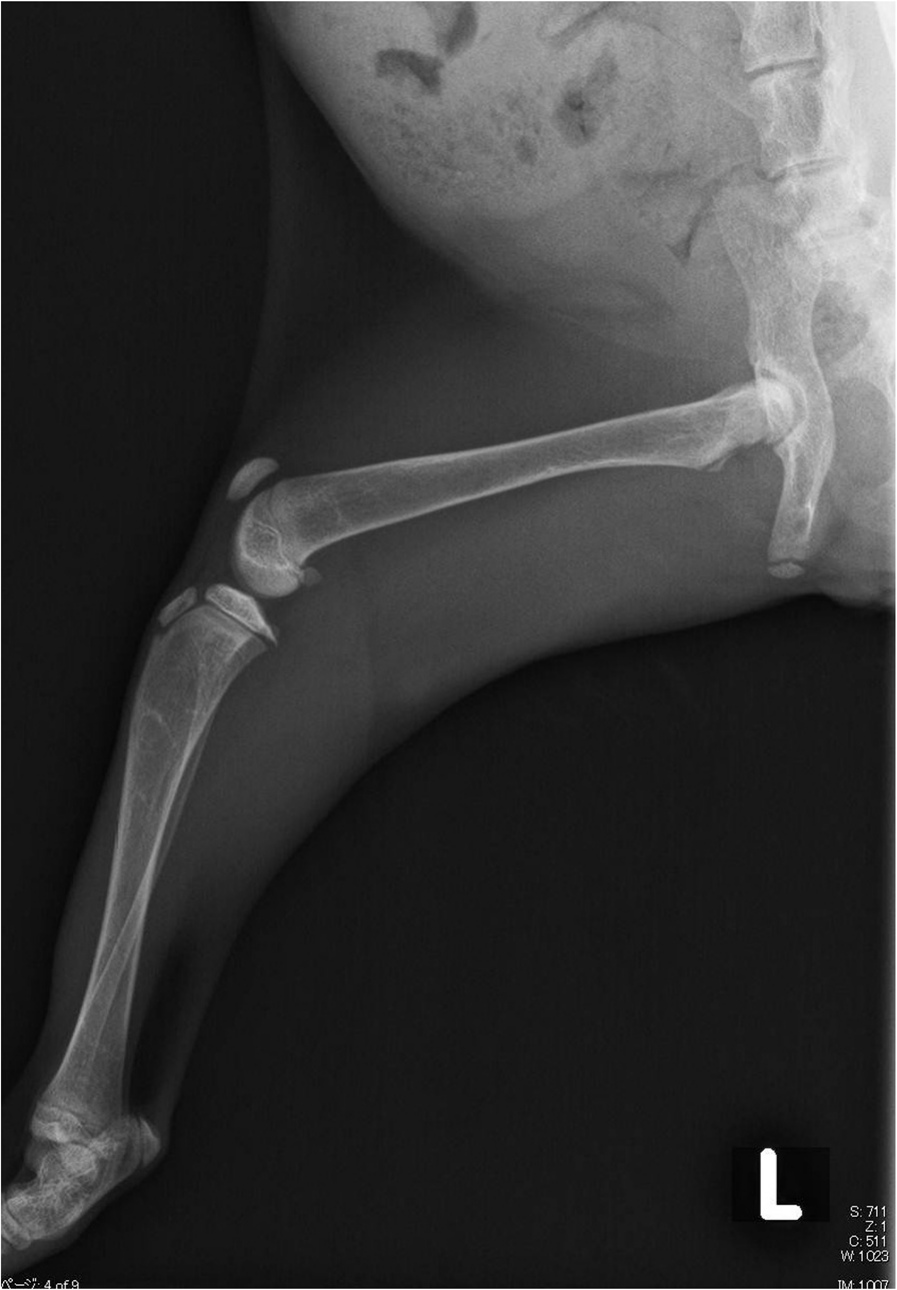
Radiograph of the left hindlimb 2.5 month after treatment

Starting on day 2, the cat received alfacalcidol (Onealfa Tablets) by CRI of 8.5% calcium gluconate. However, she did not respond well to treatment with the standard dose (0.01–0.03 μg/kg, PO, SID) of alfacalcidol so the CRI of 8.5% calcium gluconate was stopped. The final dose of alfacalcidol was very large, and 1 μg/kg, PO, TID was required to maintain the cat’s general condition in the absence of CRI of 8.5% calcium gluconate (Table 3). On day 16, the cat was discharged from hospital. Radiographs were taken again 2.5 months after the initial treatment was started. The cat’s bone structure was still abnormal, but showed marked improvement (Fig. 3).

**Fig. 4:**
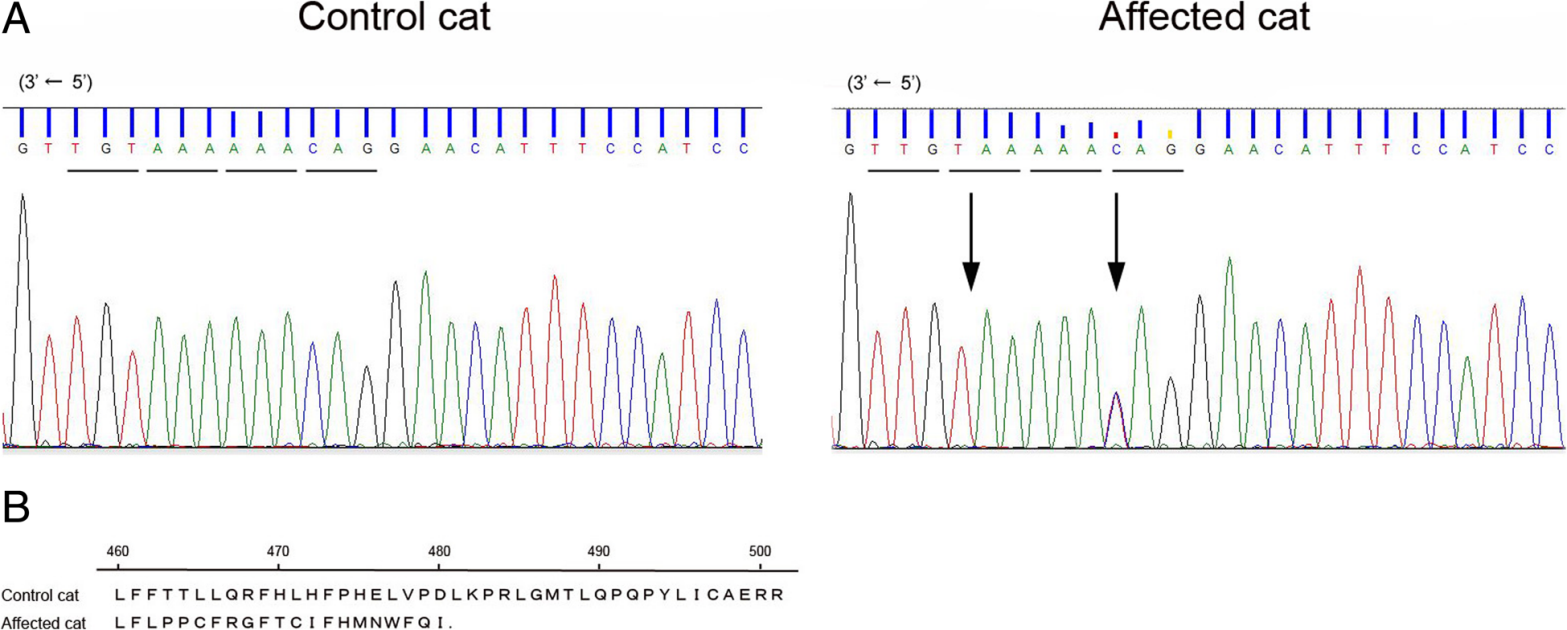
**a** A portion of the DNA sequence chromatogram *CYP2R1* exon 5 (shown 3′ → 5′ sequence), which shows the T deletion at nucleotide position c.1386 (arrow) and the heterozygous variant at position c.1380 (arrow). **b** Protein sequence alignment. The normal protein has 501 amino acids, but the sequence from the affected cat is altered from position 462 on, and a stop codon at position 481 truncates the protein

The hypocalcemia, low 1,25-dihydroxycholecalciferol, radiographic changes, and poor response to alphacalcidol led us to suspect a genetic abnormality of vitamin D metabolism in this cat. In humans and cats, type 2 VDDR is characterized by high 1,25-dihydroxycholecalciferol levels. In the cat examined in the present study, 1,25-dihydroxycholecalciferol was lower than in healthy cats, and a huge dose of alfacalcidol was required to maintain her serum calcium concentration. Hence, we decided to analyze the gene sequences of *CYP2R1* (ENSEMBL Gene ID ENSFCAG00000011383) and *CYP27B1* (ENSEMBL Gene ID ENSFCAG00000014701) in healthy control cats (2 intact males and a spayed female, all domestic short-haired cats owned by our veterinary teaching hospital staff) and the affected this cat. The feline *CYP2R* gene contains five exons encoding an enzyme of 501 amino acids, and the *CYP27B1* gene contains nine exons encoding an enzyme of 508 amino acids. We designed primer pairs for polymerase chain reaction (PCR) amplification of the individual exons and the immediate flanking regions of *CYP2R1* and *CYP27B1*. Genomic DNA was extracted from the blood samples with the DNeasy Blood & Tissue Kit (Qiagen). PCR amplification of the fragments was performed with 20 ng of genomic DNA in GoTaq Green Master Mix (Promega), which also contained the primer pair (10 μM each) and water, with 35 cycles of 30 s at 95 °C, 30 s at 55 °C, and 1 min at 72 °C (Table 4). The PCR product sizes were verified as correct by agarose gel electrophoresis separation, the products were prepared for sequencing using the FastGene Gel/PCR Extraction Kit (Nippon Genetics), and then sequenced by a commercial DNA sequencing service (Fasmac). The sequences from the healthy cats and the affected cat were compared, and although no abnormalities in the *CYP27B1* gene were present in the affected cat, we did identify the *CYP2R1* variant in exon 5; c.1380G > G/A and c.1386del in this cat (Fig. 4). The variant, where T is deleted at position c.1386, causes a frameshift mutation in the gene, resulting in chain termination at the position encoding amino acid 481 in the full-length 501 amino acid protein (p.Phe462Leufs*20). The partial sequence of CYP2R1 exon 5 in this cat has been available in the DDBJ/EMBL/GenBank databases under the accession number LC424161. We next searched for the structural information for proteins affected by this nonsynonymous mutation using the protein database HOMOCOS (http://homcos.pdbj.org/). The amino acid sequences between human and feline CYP2R1 were almost identical (96.7% identity) and all contact sites with vitamin D3 were conserved (Additional file 1). Therefore, we referred an already-known crystal structure of human CYP2R1-vitamin D3 complex (pdb ID 3c6g) [10]. Two contact sites (amino acids 487 and 488) in the CYP2R1-vitamin D3 complex were missing in the sequence from the affected cat (Additional file 2), suggesting that the T deletion in exon 5 of CYP2R1 linked with a remarkable loss of CYP2R1 protein function in this cat.

**Table 4:** PCR primer pairs used to amplify the feline CYP2R1 and CYP27B1 genes

PCR Product	Forward Primer (5′-3′)	Reverse Primer (5′-3′)
CYP2R1		
exon 1 exon 1	GCACTGTAGACTCTGAGCGG	TCACTTGCACAGAGGGTTGG
exon 2 exon 2	AGAGCTGGACTTAGCACATG	TGATTCAAGTGTGCATGCAC
exon 3 former	TGAGTAGCAGAGAACAGGGC	GCATCAACGAAATGCTGAGGT
exon 3 latter	AGTGCCTCCGTCTTCCTGTA	AGAAACTGGCGTTCAGGTCC
exon 4 exon 4	CTGGTTGTCCCCATACCCTG	CGTCCTGAAGTTATGCCCCA
exon 5 exon 5	CACTGAGCTGAAGTGGGACA	CATCCACGGCCCTTCTTACT
CYP27B1		
exon 1 exon 1	AGAGGGGGCGTCACCACTATCC	CCGGCAGCCCCAATTTCTCTAT
exon 2 exon 2	CTTGCACAGCCCAGCGAACACC	GGAAGCCCCATTTACCAGAAGG
exon 3 exon 3	CCGGGGCAGGAAATGAGTAAC	CAGGCAGCCCAGGCGTGAACC
exon 4 exon 4	GGGATGTGGCGGGAGAGTTTTA	TGGGGAGAAGGTACAGGGATGC
exon 5 exon 5	CCTGGAGGCCGAAGTGC	CCAACAGGGAGGAGAACC
exon 6 exon 6	GAGCCCAACTTCCAGAGC	CCAGGCCTTCCCAGCATTTT
exon 7 exon 7	AAGGCGGTGGTCAAGGAAGTG	CAGGCCAAAGGGAGGGTGAA
exon 8	TCCAAAAGTAGCCCCAGAA	TATTTTGCTATGCTTGTCAGAA
exon 9	TCGACCAACCTAACACTAATAA	AGAAAGGATGAGCAGACACA

After the *CYP2R1* variant was identified, the affected cat was treated with a standard dose of calcitriol (Caldemin; 2.5–3.5 ng/kg, PO, SID), which restored the total and ionized calcium levels to within the reference range. The level of 25-hydroxycholecalciferol at − 80 °C stored serum sample of initial presentation was additionally measured using a commercial ELISA kit (HVD3 ELISA Kit, MyBioSource). The low level of 25-hydroxycholecalciferol (12 nmol/L) compared with the levels of healthy cats (Table 2) is likely to type 1B VDDR in humans [11–13].

## Discussion and conclusions

To the best of our knowledge, there have been no reports of the *CYP2R1* variant seen in this study in cats or in veterinary medicine generally. In humans, a genetic defect in *CYP2R1* was first reported as a cause of rickets in 1994, in two brothers of Nigerian descent [14]. Both had rickets associated with low baseline 25-hydroxyvitamin D3 levels. Their 25-hydroxyvitamin D3 concentrations were restored to the normal range with a relatively high dose of vitamin D. The older sibling was homozygous for the transition variant c.296 T > C in exon 2 of the *CYP2R1* gene, which changes a leucine at position 99 in the encoded protein to a proline (p.Leu99Pro) [11]. A different study of familial rickets in Nigerians identified both homozygous and heterozygous c.296 T > C variants (which results in p.Leu99Pro), while the heterozygous c.726A > C nucleotide variant these researchers identified was found to result in p.Lys242Asn. Type 1B VDDR in the above-mentioned siblings resulted from a variant of *CYP2R1* [12]. Type 1B VDDR characteristically manifests as rickets in patients who fail to respond to a standard dose of vitamin D, as shown by an appropriately increased 25-hydroxyvitamin D3 levels [2]. A more recent study on type 1B VDDR in human patients described the genetic variants in *CYP2R1* as homozygous loss-of-function mutation, and found that calcifediol treatment brought about a substantive improvement in these patients [15].

After we identified the genetic mutation in *CYP2R1*, the affected cat was successfully treated with a standard dose of calcitriol. Because alfacalcidol is a prodrug, it must undergo hepatic 25-hydroxylation by CYP2R1 to become the active form, calcitriol. Hence, the total serum calcium level in the affected cat was maintained within the reference range through a standard dose of calcitriol rather than an overdose of alfacalcidol. From our examination of the crystallographic structure of human CYP2R1 complexed with vitamin D3, it is clear that the frameshift mutation in this gene will lead to an abnormal CYP2R1 protein structure in the affected cat, and this will affect the contact sites for vitamin D3. Therefore, the binding ability between CYP2R1 and vitamin D3 would be lost or at least very much reduced, thereby impairing 25-hydroxylation activity in the cat.

In conclusion, VDDR is a rare vitamin D-related deficiency disease, and type 1B VDDR has never been reported in a cat before now. The affected cat from this study will need to be treated with vitamin D3 supplementation throughout its lifetime and its serum calcium concentration will require close monitoring. It is recommended that vitamin D-dependent rickets is suspected when a poor serum calcium concentration response is seen despite administering alfacalcidol at high dosage.

## Additional files

Additional file 1: Amino acid sequence alignment of human and feline CYP2R1 proteins. The amino acid sequence identity score is 96.7% (JPG 279 kb)

Additional file 2: (A) DNA sequence alignment of exon 5 in *CYP2R1* from a control cat and the affected cat. (B) Amino acid sequence alignment of CYP2R1 from a control cat and the affected cat. The CYP2R1 protein from the affected cat is mutated from amino acid position 462 to 501. (C) Protein crystallography of CYP2R1 based on the structure of human CYP2R1 complexed with vitamin D3. Two contact sites for vitamin D3 in the protein (positions 487 and 488) are deleted. (JPG 200 kb)

## Abbreviations

CRI: Continuous rate infusion
CYP27B1: 1-alpha-hydroxylase
CYP2R1: vitamin D 25-hydroxylase
VDR: Vitamin D receptor, VDDR: vitamin D-dependent rickets
