# Supplementary figures and images for: A genetic variant of CYP2R1 identified in a cat with type 1B vitamin D-dependent rickets: a case report
Source: BMC Vet Res. 2019 Feb 18;15:62. doi: 10.1186/s12917-019-1784-1 (PMC6378717; doi:10.1186/s12917-019-1784-1)

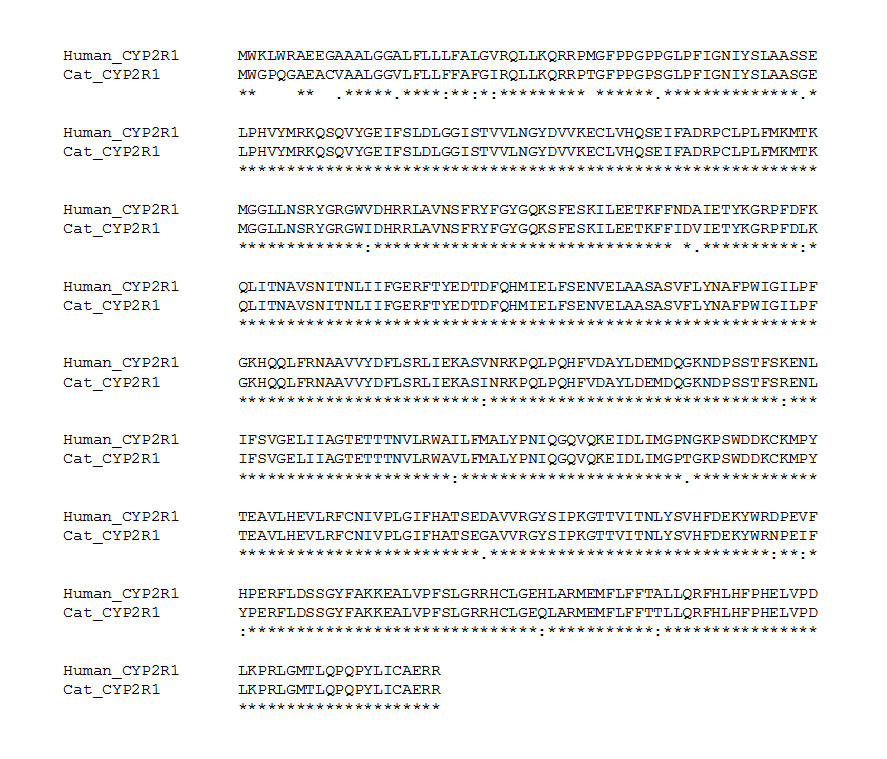

Supplement: Supplementary file 1 — Amino acid sequence alignment of human and feline CYP2R1 proteins. The amino acid sequence identity score is 96.7% (JPG 279 kb) [file 12917_2019_1784_MOESM1_ESM.jpg]

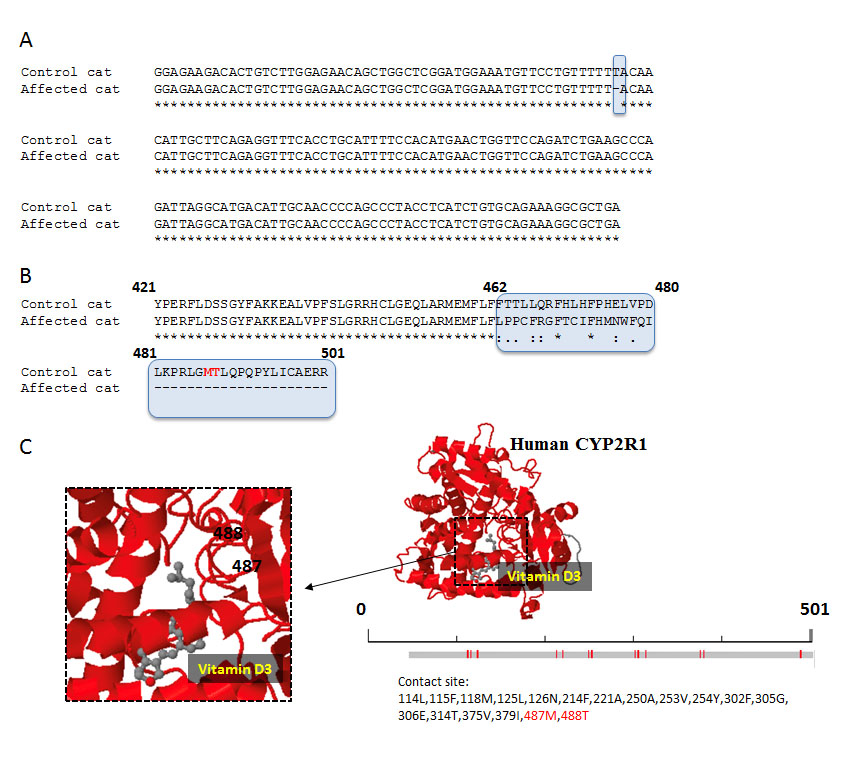

Supplement: Supplementary file 2 — (A) DNA sequence alignment of exon 5 in CYP2R1 from a control cat and the affected cat. (B) Amino acid sequence alignment of CYP2R1 from a control cat and the affected cat. The CYP2R1 protein from the affected cat is mutated from amino acid position 462 to 501. (C) Protein crystallography of CYP2R1 based on the structure of human CYP2R1 complexed with vitamin D3. Two contact sites for vitamin D3 in the protein (positions 487 and 488) are deleted. (JPG 200 kb) [file 12917_2019_1784_MOESM2_ESM.jpg]
